# Exon-Skipping Strategy by Ratio Modulation between Cytoprotective versus Pro-Apoptotic Clusterin Forms Increased Sensitivity of LNCaP to Cell Death

**DOI:** 10.1371/journal.pone.0054920

**Published:** 2013-02-13

**Authors:** Abdellatif Essabbani, Luis Garcia, Maria Josè Zonetti, Tommaso Fisco, Sabina Pucci, Gilles Chiocchia

**Affiliations:** 1 Inserm, U1016, Institut Cochin, Paris, France; 2 Université Paris Descartes, CNRS (UMR 8104), Sorbonne Paris Cite, Laboratoire d’excellence INFLAMEX, Paris, France; 3 Université Versailles-Saint-Quentin, Versailles, France; 4 Université Pierre et Marie Curie, Inserm UMR S 974, CNRS UMR 7215, Institut de Myologie, Groupe Hospitalier Pitié-Salpêtrière, Paris, France; 5 Department of Biopathology University of Rome “Tor Vergata”Policlinico”, Rome, Italy; University of Nebraska Medical Center, United States of America

## Abstract

**Background:**

In prostate cancer the secreted form of clusterin (sCLU) has been described as an anti-apoptotic protein whose expression is increased after therapeutic intervention, whereas, the nuclear protein form nCLU was reported to have pro-apoptotic properties.

**Methodology:**

In order to provide new therapeutic approaches targeting CLU, we developed a strategy based on exon skipping by using a lentiviral construct to preferentially induce the nuclear spliced form of the protein. The molecular construct was transduced in LNCaP cells for testing the modulation of sensitivity of the transduced cells to pro-apoptotic stress.

**Results and Conclusions:**

We showed an increase of nCLU/sCLU expression ratio in the prostate cancer cell line “LNCaP” after lentiviral vector-U7 nCLU transduction. Moreover, we showed a significant inhibition of cell proliferation in nCLU-U7 LNCaP cells after treatment with cisplatin and after exposure to ionizing radiation compared to control cells. Finally, we showed that nCLU-U7 LNCaP cells exposure to UV-C significantly reduced an increase of cell death compared to control. Finally, we showed that modulating nCLU expression had profound impact on Ku70/Bax interaction as well as Rad17 expression which could be a key mechanism in sensitizing cells to cell death. In conclusion, this is the first report showing that increasing of nCLU/sCLU expression ratio by using an “on demand alternative splicing” strategy successfully increased sensitivity to radiotherapy and chemotherapy of prostate cancer cells.

## Introduction

Prostate cancer is one of the most common cancers in men and the second leading cause of cancer death in men, with large differences between countries. Prostate cancer is primarily a disease of advanced age. This pathology can be the consequence of several risk factors encompassing genetic and environmental factors. To date several therapeutic interventions are proposed. The most common treatments are radical prostatectomy, radiotherapy, transperineal brachytherapy, cryotherapy, high-intensity focused ultrasound (HIFU), androgen-deprivation therapy, and chemotherapy, the latter being almost always a salvage therapy for advanced disease.

It has been shown that up-regulation of multiple gene expression is correlated to the development of prostate cancer. Among these genes, clusterin (*clu*) is considered a key factor and thus a key target. CLU is a ubiquitously expressed glycoprotein involved in several physiological functions, which includes tissue remodeling, cell-substrate interaction, lipid transport, the maturation of sperm and tissue damage [1], [2]. CLU has an important role in tumorogenesis and progression of human cancers. We and others showed that CLU is involved in the NF-κB pathway regulation [3], [4]. *Clu* transcription is complex, generating various transcript sizes and producing different cellular compartment protein forms. A secreted form (sCLU) is translated from the mRNA and includes the 9 gene exons. It is detected as an 75- to 80-kDa glycosylated protein, composed of 449 amino acids, which is cleaved into α and β subunits [5]. Experimental and clinical studies support the hypothesis that clusterin expression has a protective role against apoptotic cell death. Studies show that introduction of sCLU cDNA into LNCaP prostate cancer cells increases resistance to tumor necrosis factor (TNF) treatment induced apoptosis [6] and oxidative stress [7]. sCLU is thought to have a cytoprotective and chaperone role [8]. It is shall to play an important cytoprotective role by enhancing cellular interactions and membrane integrity of numerous cells like the pancreatic islet [9]. In addition CLU has an essential role in chemoresistance by interacting with activated Bax, thereby inhibiting cytochrome c release and apoptosis [10]. To summarize, sCLU is defined in prostate cancer as an anti-apoptotic protein whose expression is increased after radiotherapy or chemotherapy. The increased expression of sCLU appears to be correlated to the drug resistance to treatment and cancer progression. The very mechanism of action through which sCLU allows cell survival is not yet fully elucidated.

A *Clu* gene isoform resulting from alternative splicing of exon 2 mRNA has also been reported [11]. This form is detected as a 49 kDa nonglycosylated precursor nCLU protein (pnCLU) in the cytosol and a 55 kDa glycosylated protein (nCLU) in the nucleus. pnCLU is induced and translocated from the cytoplasm to the nucleus in response to cell damage and several cytotoxic events, including ionizing radiation (IR) [11], [12]. This “translocation” of nCLU could be permitted by using the NLS (Nuclear Localization Signal) sequence identified in exon 3. Conversely to sCLU several reports demonstrated that nCLU could be a pro-apoptotic protein. Along this line, nCLU can bind to the DNA binding protein KU-70 [13], which is involved in double-stranded DNA repair and cooperates with Ku70 to induce apoptotic death after an irreversible cell damage, activating the translocation of BAX to mitochondria. In particular it was demonstrated that nCLU could serve as a chaperone to conduct KU70. Thus, nCLU protein is believed to be a proapoptotic protein, with sCLU antagonistic activity. The CLU/KU-70 interaction might directly affect NHEJ (non-homologous end joining) DSB (double strand break) repair processes. In prostate cancer it has already been shown that overexpression of nCLU in cells led to the G2-M phase arrest and caspase dependent apoptosis [14]. Another study showed the accumulation of nCLU in prostate cells following proapoptotic stimuli [15]. It has already been demonstrated that silencing expression of the clusterin gene in cancer cells using small interfering RNA induces spontaneous apoptosis, reduced growth ability, and cell sensitization to genotoxic and oxidative stress [16], [17]. Furthermore, a previous study showed that the inhibition of CLU expression by use of antisens oligonucleotide (OGX-11) reduced expression of that protein. The combination between OGX-11 and chemotherapy demonstrated improved treatment of prostate cancer compared to separate treatment [18].

In light of CLU expression knowledge and its role in cancer, we sought new methods allowing down-regulation of the sCLU form while increasing expression of the nCLU form. To reach this goal we developed an “on demand alternative splicing” strategy based on the use of forced exon skipping.

## Results and Discussion

### U7-nCLU Vector and Induced nCLU Expression

CLU has recently received a lot of attention because of its association with cancer. Interestingly, two different isoforms of CLU appear to be involved in either prosurvival or apoptosis processes with sCLU being a prosurvival factor whereas nCLU a proapoptotic trigger [19]. The fate of prostate cancer cells seems to depend on the sCLU/nCLU ratio. Hence, CLU appeared to be a good target in cancer and modulating the ratio sCLU/nCLU by increasing nCLU and/or down-regulating sCLU appeared promising.

**Figure 1:**
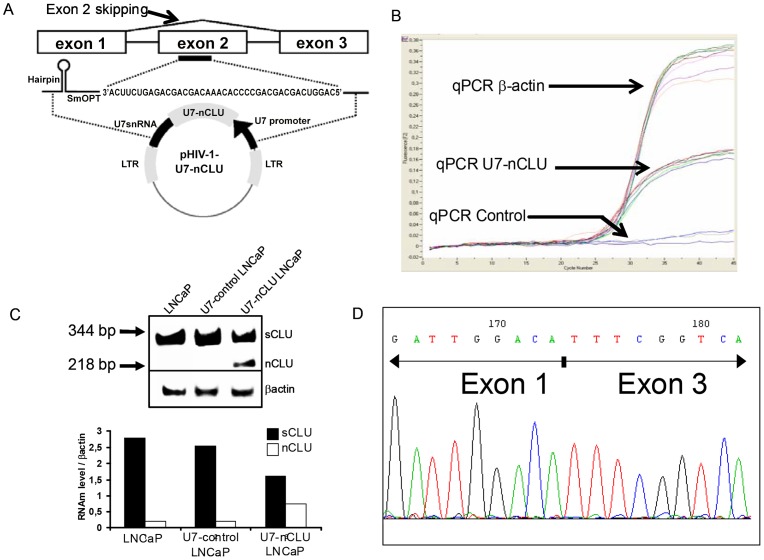
U7-nCLU vector construct and CLU mRNA expression in LNCaP. (A) (Top) Antisens sequence position to induce exon 2 skipping. (Bottom) Structure of the pHIV-1-U7-nCLU. U7snRNA including antisens sequence is placed under the control of its own promoter and its regulating domains 3′. SmOPT: sequence indicates the Sm protein binding site. LTR : long terminal repeats. (B) U7 vector integration in transduced LNCaP cell genome. Curves represent qPCR amplification of U7-nCLU genomic DNA in different transduction conditions (middle curves). Human β-actin amplification corresponded to positive control (Top curves) and genomic DNA of non transduced cells was used as negative control. (C) (Top) mRNA from transduced LNCaP cells with U7-nCLU vector was analyzed by RT-PCR at 3 weeks posttransduction. The 344 bp and 218 bp bands correspond to sCLU and skipped form nCLU mRNA respectively without alteration of the sequence. Controls were from RNA of LNCaP alone or LNCaP transduced with U7-control. (Bottom) Relative mRNA expression levels determined by densitometry. βactin used to normalize changes in specific gene expressions. (D) DNA sequence of the 218 bp band showed skipping of exon 2.

To carry out our research we used an exon skipping strategy based on antisens oligonucleotide that removed the targeted exon. The selected sequence covered a specific ESE (exon splice enhancer) region of exon 2 which is involved in exon definition during the CLU pre-mRNA splicing process [20] (Figure 1*A*). Removing exon 2 during the CLU mRNA maturation preferentially generated the nCLU isoform. The antisense sequence was incorporated into an optimized small nuclear U7 RNA (U7 snRNP) to allow sustained expression (Figure 1*A*). This strategy has already proven very effective *in vivo* in the mdx mouse using a vectorized cassette in both Adeno Associated Virus- and lentivirus-based vectors [21], [22]. In our construct, the optimized U7 carrying the antisense sequence was inserted encompassing the woodchuck hepatitis virus responsive element (WPRE) and the central polypurine tract (cPPT). This construct was then encompassed into HIV-1 derived vectors resulting in a major improvement of lentiviral vector performance (Figure 1*A*).

To test the construct vector expression, we chose a prostate cell line (LNCaP) model, which expresses almost exclusively the sCLU form compared to nCLU [7]. Vector genomic integration was confirmred by qPCR (Figure 1*B*) and subsequent expression of clusterin isoforms (sCLU/nCLU) in different transduced cell lines was assessed by Reverse Transcription *-* Polymerase Chain Reaction (RT-PCR). As expected, parental LNCaP cells as well as cells transducted with a lentivector harbouring an irrelevant antisense sequence linked to U7 (U7-control) expressed mainly the sCLU mRNA compared to nCLU. Conversely, LNCaP cells transduced with U7-nCLU vector expressed both CLU isoforms. With an increase of nCLU and a decrease of sCLU expression resulting on thereabouts 18 to 20 fold variation of the nCLU/sCLU ratio was seen (Figure 1*C*, top and bottom). Accurate skipping of exon 2 was checked by direct sequencing the RT-PCR end products (Figure 1*D*).

### nCLU-U7 Transduction Sensitized LNCaP Cells to Cisplatin Drug Treatment

Several studies showed that CLU (likely sCLU) expression increased after cisplatin treatment and that down regulation of sCLU enhanced the cisplatin-induced chemosensitivity in many type of cancers including prostate cancer [23]. Nevertheless, few or no data are available regarding the role of nCLU form in this drug response. To test nCLU induction expression effect in response to cisplatin treatment, we analysed the proliferative response of nCLU-U7 transduced LNCaP cells after different cisplatin-dose treatments.

**Figure 2:**
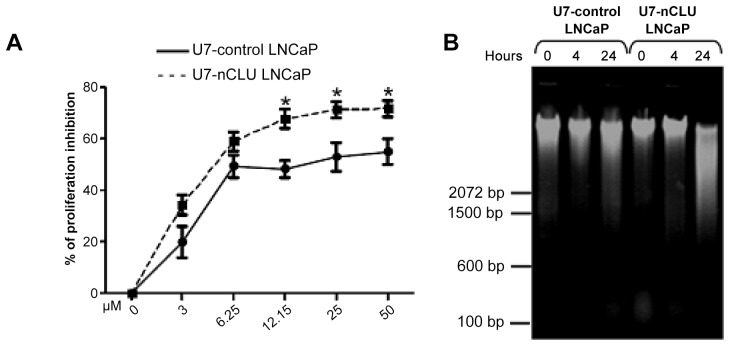
Effect of cisplatin drug on nCLU-U7 LNCaP cells. (A) LNCaP cells were treated at the indicated doses for 24 hours and proliferation was assessed. (*): P value <0.05 (Student’s t-test) vs control cells. (B) Effect of cisplatin treatment on DNA fragmentation of LNCaP cells. LNCaP cells were treated with 50 µM cisplatin for 0, 4 and 24 hours. Cellular DNA was isolated and subjected to agarose gel electrophoresis.

These results showed that cisplatin treatment of 12.5 µM, or great, for 24 h resulted in a profound and significant reduction of proliferation of U7-nCLU LNCaP compared to U7-control LNCaP (Figure 2*A*).

To determine whether U7-nCLU transduction enhanced cisplatin–induced DNA fragmentation of LNCaP cells, the cells were treated for 24 h by ciplastin and DNA fragmentation was analysed. The DNA fragments typical of apoptosis were observed only in U7-nCLU transduced LNCaP compared to control (Figure 2*B*). This result was reminiscent of the results of Chung et al. showing that the inhibition of clusterin expression of bladder cancer cells by anti-sense transfection enhanced cisplatin-induced cytotoxicity accompanied by DNA fragmentation [24].

### Modulation of LNCaP Cells Sensitivity to Ionizing Radiation Following Transduction with nCLU-U7 Vector

CLU has been described as a cell survival gene in radiation-induced cell death in human LNCaP prostate cancer cells [25]. On the other hand, nCLU is an ionizing radiation (IR)-inducible protein that binds Ku70, and triggers apoptosis when overexpressed in MCF-7 cells [11]. Thus, it was important to compare radiation sensitivities in parental and U7-nCLU LNCaP cells.

**Figure 3:**
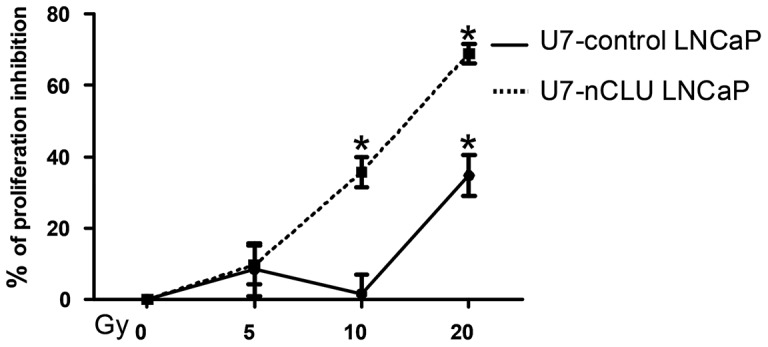
Increase sensitivity of LNCaP cells to irradiation following U7-nCLU LNCaP transduction. IR dose exposure effect at 48 hours on LNCaP cells proliferation. Data are expressed as % inhibition of the mean counts per minute (CPM) from 3 individual experiments. (*): P value <0.05 (Student’s t-test) vs control cells.

In this context we observed that an IR of 10 Gy significantly induced a 40% reduction of proliferation in U7-nCLU LNCaP compared to less than 5% in U7-control LNCaP (Figure 3). Furthermore, at 20 Gy the inhibition of proliferation observed was twofold higher in U7-nCLU LNCaP (70%) compared to control (35%).

These findings demonstrated that enforced nCLU expression in LNCaP cells resulted in an increase of the nCLU/sCLU ratio thereby according to our hypothesis, improving the sensitivity of these cells to IR.

### U7-nCLU Vector Expression Increased UV-induced LNCaP Cell Death

To further evaluate the effect of modulating the nCLU/sCLU ratio in LNCaP cells, we used an UV-C irradiation technical [26] and we followed-up cell death and apoptosis of treated cells.

**Figure 4:**
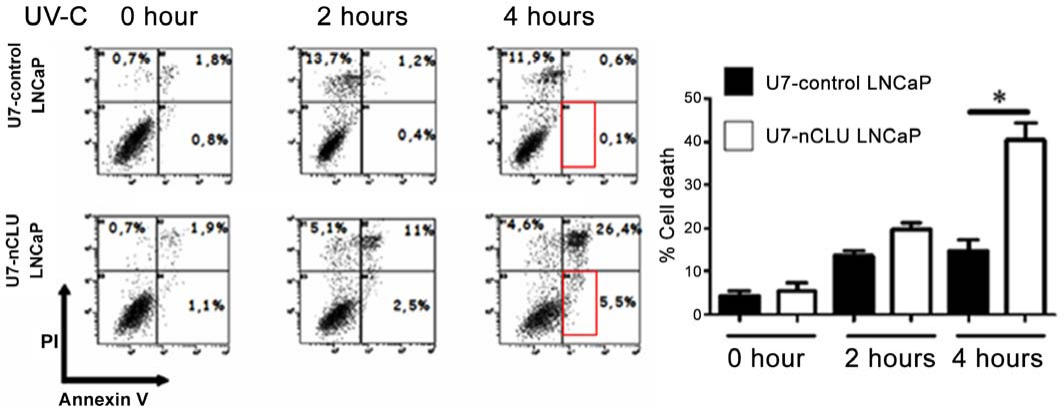
Increased UV-C induced death following U7-nCLU vector expression in LNCaP cells. FACS analysis was performed at 0, 2 and 4 hours after 10 min UV-C cell exposure. Cell death was assessed using propidium iodide (PI)/annexin V staining. The red rectangle surrounds PI−/Annexin V+cells. Histogram represent % of dead cells in each condition. Results are means +/− s.e.m. from 3 individual experiments. (*): P value <0.05 (Student’s t-test).

We observed an increase in cellular death in U7-nCLU LNCaP compared to U7-control LNCaP as soon as 2 hours post-UV-C radiation. This difference became even more important and significant at 4 h (Figure 4). Moreover these results showed at least part of U7-nCLU LNCaP cells may undergo apoptosis (5.5%) compare to control (0.1%) (Figure 4, see red rectangle, using PI/annexin V staining).

### nCLU Expression Modulate Bax/Ku70 Interaction and Rad17 Expression

**Figure 5:**
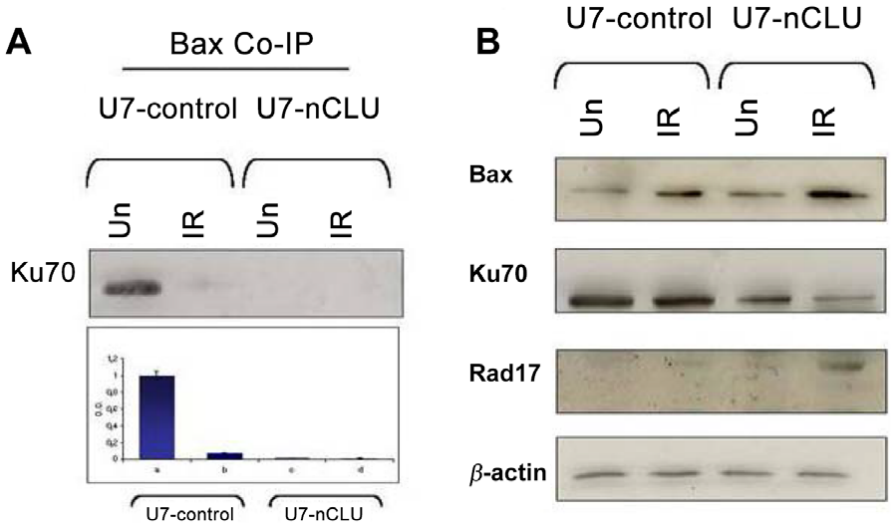
nClu expression led to defective interaction between Bax and Ku70. (A) Coimmunoprecipitation of Bax and Ku70 following or not irradiation. No Bax- Ku70 coimmunoprecipitation was found at time 0 in not irradiated U7-nClu cells compare to U7 control cells. No coimmunoprecipitation of Bax and Ku70 was observed in all irradiated cells both at 4 hrs (not shown) and 24 hrs. (B) Defective expression of KU70 in nCLU-transduced cells. Note the upregulation of Rad17 protein following irradiation of nCLU-LNCap.

In order to examine the dynamic interactions among Bax and Ku70 in the presence of a constitutive expression of nCLU in the U7-nCLU transduced cells as compared to the U7-control, Bax co-immunoprecipitation was performed on total proteins extracted from unirradiated or irradiated cells. As shown in Figure 5 in unirradiated U7-control cells, Ku 70 co-immunoprecipitated with Bax (Figure 5*A*) in agreement with its inactive status in the cytoplams in absence of damage. At time 24 hrs post irradiation, in same experimental group, Ku70 was almost totally released from Bax allowing its dimerization and translocation in the mitochondria. Conversely in both untreated and irradiated U7-nCLU cells no Ku70-Bax interaction was found as shown in Figure 5*A*. This result indicated that the constitutive expression of nCLU isoform interferes with Ku70-Bax induced inactive state leading to a prompt response to cell damage. These results confirmed data on higher apoptotic cell percentage after irradiation in U7-nCLU LNCaP transducted cells. In addition, Bax, Ku70 and Rad17 protein levels were examined by Western blot 24 hrs after irradiation (Figure 5*B*). In that case, we observed a strong increase of Bax level and a concomitant conspicuous decrease of Ku 70 expression in irradiated cells especially in nClu transduced cells in respect to U7-control cells. Conversely, at 24 hrs, a strong increase of Rad 17 protein, primarily involved in radiation-induced G2/M checkpoint of cells, was observed in U7-nCLU transduced cells as compared to WT irr after 24 hrs. Presently, we have no direct evidence that nCLU somehow chaperones or stabilizes the Ku70-Bax cytosolic interaction. Of note, the loss of Ku70-Bax binding in nCLU over-expressing cells we observed might as well be an indirect effect due to the nCLU-mediated triggering of apoptosis.

### Concluding Remarks

Herein, we demonstrated the possibility to modulate sCLU/nCLU ratio using a new approach, by mean of exon skipping that removes the targeted exon 2 of *clu* mRNA. We described a lentiviral construct allowing potent modulation of this ratio (up to 20 fold). Then we studied the fate of prostate cancer cells following different type of stress after lentiviral transduction. We demonstrated that increasing the amount of nCLU resulting in a strong decrease of the sCLU/nCLU ratio reduced growth ability without inducing spontaneous apoptosis of transduced cells and increased drastically LNCaP cells sensitization to genotoxic and ionizing irradiation stress by apoptosis. Interestingly, we observed that U7-nCLU transduced LNCaP cells have also been sensitized to UV-induced cell death by apoptosis although it is yet unclear if this type of cell death only could fully explained the enhanced cell death of the cells. The constitutive expression of the proapoptotic nCLU isoform, usually strongly induced after cell damage, displays an antagonistic role in respect to the endogenous sCLu responsible of radio- and chemio resistance. In conclusion results obtained suggest that nCLU overexpression could be useful to sensitizes and predispose prostate cancer cells to apoptotic process, conferring a greater efficiency in many anticancer strategies that activate pro-survival pathways and the formation of resistant phenotypes. Furthermore, whatever the level of clusterin expression, the use of exon-skipping approach we report could be usable because it consists in redirecting the clu expression of the cells and not to shut it down.

## Materials and Methods

### U7-nCLU Lentiviral Vector Construction

Antisense sequence targeting clusterin exon 2 was obtained by using ESE Finder software. U7-nCLU construct was engineered from the previously described U7smOPT-SD23/BP22 [16] and then introduced in lentiviral vector construct pRRL-cPPT-hPGK-eGFP-WPRE. The control corresponded to murin BAFF antisens sequence introduced in a lentiviral vector.

### Cell Culture and Conditions

Human LNCaP cells were grown in RPMI 1640 supplemented with 5% heat-inactivated fetal calf serum and 1% penicillin-streptomycin (Invitrogen) at 37°C in a humidified atmosphere of 5% CO_2_. For transduction, the cells were transduced at the multiplicity of infection of hundred lentiviral constructs for one cell. The cells were used from 2 weeks post-transduction following test of construct expression and efficiency by western blotting and real-time PCR.

### Real-time PCR Analysis

Genomic DNA was subjected to quantitative PCR using a LightCycler system (Roche Diagnostics) according to manufacturer instructions. Two pairs of primers were used for the reactions: one pair for the amplification of U7-nCLU sequence, and a pair for amplification of β-actin (used as control).

U7-nCLU forward: 5′-CACTGACAATTCCGTGGTGT-3′ U7-nCLU reverse: 5′-AATCCAGGTGGCAACACAG-3′ β-actin forward: 5′-GGGTCAGAAGGATTCCTATG-3′ β-actin reverse: 5′-GGTCTCAAACATGATCTGGG-3′.

Probe specific amplicons expected for nCLU U7-and β-actin were stained by fluorescein (FAM)-labeled hydrolysis probes obtained from the Universal ProbeLibrary assay (Roche Applied Science). Final results were quantified using the software Rel-Quant (Roche Diagnostics).

### LNCaP Cell Treatment and Proliferation

LNCaP cells were plated in 96-well plates at a seeding density of 5×10^3^ and treated with cisplatin or exposed to irradiation (^137^Cs gamma rays) at the indicated doses.

Proliferation of LNCaP cells was assessed by measuring incorporation of 3H-deoxythymidine (0.5 µCi per well) added during the last 16 hours before harvesting, using a 1450 Microbeta scintillation counter (Wallac, Turku, Finland). Data are expressed as the mean counts per minute in octoplicate.

### Assays of DNA Fragmentation

Cells were treated with 50 µM of cisplatin at differents times. Cell lysis and DNA genomic extracts were achieved using the QIAGEN DNAeasy kit. Samples were electrophoresed on 0.7% agarose gels. DNA fragments were visualized by UV light transillumination. Photographs were taken by computer-assisted image processor (Bio-Rad Laboratories).

### Assessment of Apoptosis

Apoptosis assessment of LNCaP cells was performed using the annexin V kit (BD Biosciences). To this end, cells were exposed to UV-C and harvested 2 and 4 h post irradiation. The cells were then analysed following annexin V-FITC binding and propidium iodide uptake, accordingly to the manufacturer’s instructions. Samples were analyzed using an FC500 cytometer (Beckman Coulter, Villepinte, France), and the CxP analysis software (Beckman Coulter).

### Immunoprecipitation Assay (IP)

Immunoprecipitation assay (IP) was performed on total protein extracts from LNCap cells (U7-nCLU trasduced LNCap cells) exposed or not to irradiation for 24 hours. 100 ug of total extracts were used for immunoprecipitation experiments. Total extracts were incubated with 4 µg of anti-Bax (clone 2D2, Neomarker) in 250 ul of reaction buffer (PBS +0.05% NP-40) and incubated overnight at 4°C with rotation. Then, Protein G-Agarose (Santa Cruz Biotechnology 50% slurry) was added for 2 hours at 4°C with agitation. IP negative control was performed without specific antibody. Each immunoprecipitate was loaded on a 12% SDS-PAGE. Proteins were blotted onto PVDF membrane (Hybond-P, Amersham Biosciences), stained with Ponceau S-dye and then probed with the various antibodies reported in the Western Blot section below.

### Western Blotting

Total protein extracts (20 µg) were loaded on a 12% SDS-PAGE. Proteins were transferred to a PVDF membrane (Hybond-P, Amersham Biosciences). Membranes were probed with the following primary antibodies: anti-Ku70 (A9); anti-Bax (NeoMarker, clone 2D2); anti-Rad-17 (H300, Santa Cruz Biotechnologies, Inc.,), and anti-β-actin (Sigma-Aldrich, USA) antibodies.

## References

[1] Jones SE, Jomary C (2002) Clusterin. Int J Biochem Cell Biol 34: 427–431. 11906815 10.1016/s1357-2725(01)00155-8

[2] Falgarone G, Chiocchia G (2009) Chapter 8: Clusterin: A multifacet protein at the crossroad of inflammation and autoimmunity. Adv Cancer Res 104: 139–170. 19878776 10.1016/S0065-230X(09)04008-1

[3] Cagnard N, Letourneur F, Essabbani A, Devauchelle V, Mistou S, et al (2005) Interleukin-32, CCL2, PF4F1 and GFD10 are the only cytokine/chemokine genes differentially expressed by in vitro cultured rheumatoid and osteoarthritis fibroblast-like synoviocytes. Eur Cytokine Netw 16: 289–292. 16464743

[4] Essabbani A, Margottin-Goguet F, Chiocchia G (2010) Identification of clusterin domain involved in NF-kappaB pathway regulation. J Biol Chem 285: 4273–4277. 20028970 10.1074/jbc.C109.057133 PMC2836031

[5] Humphreys D, Hochgrebe TT, Easterbrook-Smith SB, Tenniswood MP, Wilson MR (1997) Effects of clusterin overexpression on TNFalpha- and TGFbeta-mediated death of L929 cells. Biochemistry 36: 15233–15243. 9398251 10.1021/bi9703507

[6] Sensibar JA, Sutkowski DM, Raffo A, Buttyan R, Griswold MD, et al (1995) Prevention of cell death induced by tumor necrosis factor alpha in LNCaP cells by overexpression of sulfated glycoprotein-2 (clusterin). Cancer Res 55: 2431–2437. 7757997

[7] Miyake H, Hara I, Gleave ME, Eto H (2004) Protection of androgen-dependent human prostate cancer cells from oxidative stress-induced DNA damage by overexpression of clusterin and its modulation by androgen. Prostate 61: 318–323. 15389725 10.1002/pros.20087

[8] French LE, Wohlwend A, Sappino AP, Tschopp J, Schifferli JA (1994) Human clusterin gene expression is confined to surviving cells during in vitro programmed cell death. J Clin Invest 93: 877–884. 8113419 10.1172/JCI117043 PMC293954

[9] Park IS, Che YZ, Bendayan M, Kang SW, Min BH (1999) Up-regulation of clusterin (sulfated glycoprotein-2) in pancreatic islet cells upon streptozotocin injection to rats. J Endocrinol 162: 57–65. 10396021 10.1677/joe.0.1620057

[10] Zhang H, Kim JK, Edwards CA, Xu Z, Taichman R, et al (2005) Clusterin inhibits apoptosis by interacting with activated Bax. Nat Cell Biol 7: 909–915. 16113678 10.1038/ncb1291

[11] Leskov KS, Klokov DY, Li J, Kinsella TJ, Boothman DA (2003) Synthesis and functional analyses of nuclear clusterin, a cell death protein. J Biol Chem 278: 11590–11600. 12551933 10.1074/jbc.M209233200

[12] Markopoulou S, Kontargiris E, Batsi C, Tzavaras T, Trougakos I, et al (2009) Vanadium-induced apoptosis of HaCaT cells is mediated by c-fos and involves nuclear accumulation of clusterin. Febs J 276: 3784–3799. 19531052 10.1111/j.1742-4658.2009.07093.x PMC4810032

[13] Yang CR, Yeh S, Leskov K, Odegaard E, Hsu HL, et al (1999) Isolation of Ku70-binding proteins (KUBs). Nucleic Acids Res 27: 2165–2174. 10219089 10.1093/nar/27.10.2165 PMC148436

[14] Caccamo AE, Scaltriti M, Caporali A, D’Arca D, Scorcioni F, et al (2003) Nuclear translocation of a clusterin isoform is associated with induction of anoikis in SV40-immortalized human prostate epithelial cells. Ann N Y Acad Sci 1010: 514–519. 15033782 10.1196/annals.1299.095

[15] Caccamo AE, Scaltriti M, Caporali A, D’Arca D, Corti A, et al (2005) Ca2+ depletion induces nuclear clusterin, a novel effector of apoptosis in immortalized human prostate cells. Cell Death Differ 12: 101–104. 15499376 10.1038/sj.cdd.4401491

[16] Trougakos IP, So A, Jansen B, Gleave ME, Gonos ES (2004) Silencing expression of the clusterin/apolipoprotein j gene in human cancer cells using small interfering RNA induces spontaneous apoptosis, reduced growth ability, and cell sensitization to genotoxic and oxidative stress. Cancer Res 64: 1834–1842. 14996747 10.1158/0008-5472.can-03-2664

[17] Trougakos IP, Gonos ES (2004) Functional analysis of clusterin/apolipoprotein J in cellular death induced by severe genotoxic stress. Ann N Y Acad Sci 1019: 206–210. 15247015 10.1196/annals.1297.033

[18] Gleave M, Miyake H (2005) Use of antisense oligonucleotides targeting the cytoprotective gene, clusterin, to enhance androgen- and chemo-sensitivity in prostate cancer. World J Urol 23: 38–46. 15770517 10.1007/s00345-004-0474-0

[19] Rizzi F, Bettuzzi S (2008) Targeting Clusterin in prostate cancer. J Physiol Pharmacol 59 Suppl 9265–274. 19261985

[20] Cartegni L, Chew SL, Krainer AR (2002) Listening to silence and understanding nonsense: exonic mutations that affect splicing. Nat Rev Genet 3: 285–298. 11967553 10.1038/nrg775

[21] Goyenvalle A, Vulin A, Fougerousse F, Leturcq F, Kaplan JC, et al (2004) Rescue of dystrophic muscle through U7 snRNA-mediated exon skipping. Science 306: 1796–1799. 15528407 10.1126/science.1104297

[22] Benchaouir R, Meregalli M, Farini A, D’Antona G, Belicchi M, et al (2007) Restoration of human dystrophin following transplantation of exon-skipping-engineered DMD patient stem cells into dystrophic mice. Cell Stem Cell 1: 646–657. 18371406 10.1016/j.stem.2007.09.016

[23] Lee CH, Jin RJ, Kwak C, Jeong H, Park MS, et al (2002) Suppression of clusterin expression enhanced cisplatin-induced cytotoxicity on renal cell carcinoma cells. Urology 60: 516–520. 12350509 10.1016/s0090-4295(02)01806-x

[24] Chung J, Kwak C, Jin RJ, Lee CH, Lee KH, et al (2004) Enhanced chemosensitivity of bladder cancer cells to cisplatin by suppression of clusterin in vitro. Cancer Lett 203: 155–161. 14732223 10.1016/j.canlet.2003.07.008

[25] Zellweger T, Chi K, Miyake H, Adomat H, Kiyama S, et al (2002) Enhanced radiation sensitivity in prostate cancer by inhibition of the cell survival protein clusterin. Clin Cancer Res 8: 3276–3284. 12374699

[26] Gentile M, Latonen L, Laiho M (2003) Cell cycle arrest and apoptosis provoked by UV radiation-induced DNA damage are transcriptionally highly divergent responses. Nucleic Acids Res 31: 4779–4790. 12907719 10.1093/nar/gkg675 PMC169943

